# One Patient (and One Physician) at a Time

**DOI:** 10.4269/ajtmh.20-0051

**Published:** 2020-06

**Authors:** Tommaso Manciulli, Gaukhar Doshzanova, Aigerim Mustapayeva

**Affiliations:** 1 University of Pavia, Pavia, Italy;; 2 Asfendiyarov Kazakh National Medical University, Almaty, Kazakhstan

As I watch a sign with the name of a small hospital written in Cyrillic characters, a young nurse comes out and waves her hand telling me to go back inside the building. It is day 20 of my month in Kazakhstan, and I am working with local colleagues to conduct an ultrasound-based screening for cystic echinococcosis in small villages in South Kazakhstan Oblast (the equivalent of a region in former Soviet Union countries). I have now been in central Asia for a month and a half, first in Uzbekistan in September, and then here.

After a first round of screening in the Almaty Oblast, the Kazakh ultrasound physicians on the team are now able to process patients themselves. I decided to step out to briefly enjoy the view of the sun reflecting on the nearby mountains. I take the chance to call A. M., my main local partner. She is a young radiologist and Ph.D. student like myself, who has decided to work on cystic echinococcosis and is currently taking time off from her clinical duties to work for free on the field project. The nurse who came to call me back inside while I was on the phone is not able to tell me why she was sent out, but I can see that they found a case for our study from her look.

When I step inside one of the two rooms we are using for ultrasound examination, a young girl—she must be 14 or 15 years old—is on the small bed where patients lie as they go through a full abdominal examination. A small crowd of people greets me when I enter the room. The patient’s parents are near the door of the room, looking at the girl with worried faces as one of the Kazakh residents, Lyazzat, is performing the ultrasound. As I approach, I see that the two students from Italy who came with me are also present. Gian Luca and Agnese are staring at the screen as they try to spot the reason why I was called in the room. The senior physician for the hospital—a surgeon who called the parents when he learned our team was going to screen patients in his village—completes the picture.

**Figure 1.**
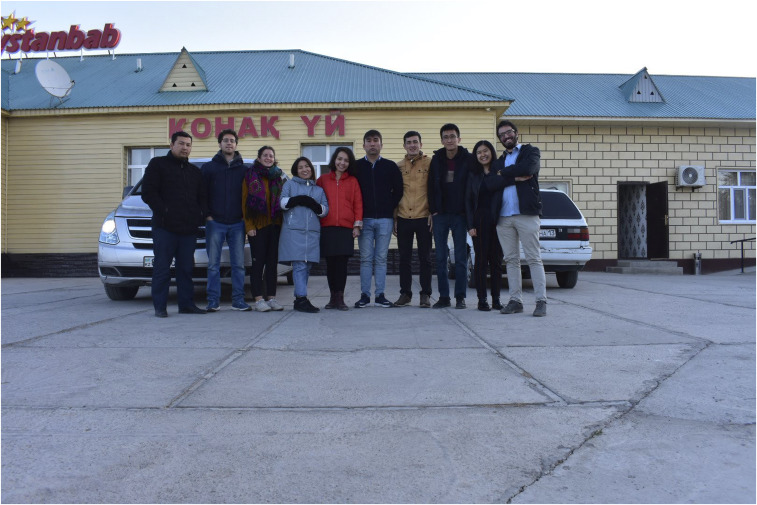
The screening team before heading into the field in the Shymkent region. This figure appears in color at www.ajtmh.org.

As I walk toward the screen, I can see why the nurse called me inside. The girl has several lesions in her liver. All the cysts appear to be inactive, and Lyazzat has already figured this out as she has proven to be a quick study during our time together. I ask her to give me her spot and do a second scan of the liver and abdomen to confirm, and count eight inactive lesions. I then ask to call the rest of the screening team, consisting of two other radiology residents from the national referral center for surgery—Erniar and Samat—Nurbol, a radiology resident working under A. M. with Lyazzat, and G. D., a pediatrician who is also a candidate to enter a local Ph.D. program (Figure 1).

Then, perhaps the hardest part begins: we need to explain what we have found to the patient and her physicians. This has been a constant problem during my time in Kazakhstan and Uzbekistan. Whenever we found inactive cysts, making people—both patients and physicians—understand that these should not be operated on was hard. To be fair, the task is sometimes difficult in Italy as well. In this specific case, we need to explain that although the lesions are many, they are small and completely solid. As such, a reactivation is unlikely. The patient should not undergo surgery.

Aided by Lyazzat and Nurbol, the group learns that the young girl has been taking albendazole for 2 years now. The senior physician tells me that he refused the surgical intervention that was initially proposed in a center in Shymkent. We then ask if she has any past images of the cysts, and we are handed a computed tomography scan showing the same number of lesions as well as the fact that these were already solid when she started albendazole. She can stop albendazole, or rather she could have avoided starting at all. The translation takes some time as the Kazakh language is complex and full of nuances. After the last word is spoken by Nurbol, and while I am still scanning my patient to save clips of the cysts, she and her parents start to cry. I immediately ask if I am pressing the probe too hard on the abdomen, but Nurbol quickly explains that they are crying out of relief. The patient is now free of having to take two pills every day and, perhaps most importantly, her treatment will no longer constitute a financial burden for the family.

Then, we go on to explain this to the surgeon, giving a quick overview of the WHO-endorsed classification of cystic echinococcosis cysts. We congratulate him for avoiding an unnecessary surgery for this patient (something which is not always done by local physicians). He understands what we have explained and asks for help on future cases. I am happy to answer that while we are there only for that day and the next, we are working with Kazakh colleagues in Almaty, and now he will have a team of local experts to refer to. He now seems to understand why these cysts did not have to be treated at all, even medically. He thanks us and exits the room to help organize the many patients waiting outside to be scanned.

I ask Nurbol to see the next patient coming in and exit the room to find the family I have just seen. They greet me with a smile. I call A. M. again to quickly inform her. She has been with us for the first round of screening and is now back in Almaty contacting doctors from the next villages. We discuss plans for the next few days, and she updates me on another case we encountered a few days back. A young woman came showing evident signs of thoracic surgery and explained that she had surgery four times but never took albendazole after each intervention. Now, she has relapsing cystic echinococcosis in her mediastinum, and we are trying to have her come to Italy for treatment. Out of desperation, she went to a private hospital where Korean doctors proposed to treat her with traditional medicine. She spent US$20,000 without any progress in the treatment.

Adherence to expert recommendations in cystic echinococcosis is scarce, which is often the cause of patient misdiagnosis and mistreatment. This is true in both developing and developed countries, where many cases such as these two can be found. Dealing with this disease is certainly frustrating for the patient, but physicians also enjoy their fair share of stress and occasional bursts of impotence. However, as I watch the now happy family leave the hospital, I do think that things can and will get better, perhaps one patient (and one physician) at a time.

Dr. T. M., M.D. is a Ph.D. candidate at the University of Pavia. He has worked at the Italian referral center for Cystic Echinococcosis in Italy for the past 4 years and is set to defend a thesis on the clinical management of cystic echinococcosis. He has been involved in ultrasound-based screenings for cystic echinococcosis in developing countries (Perù, India, Kazakhstan, and Uzbekistan).

Dr. A. M., M.D. is senior radiologist and Ph.D. student at the Kazakh National Medical University in Almaty, Kazakhstan. She is working on the epidemiology of cystic echinococcosis in Kazakhstan as the main subject for her Ph.D. thesis.

Dr. G. D., M.D. is pediatrician starting her Ph.D. studies at at the Kazakh National Medical University in Almaty, Kazakhstan. She is working on the epidemiology of cystic echinococcosis in children in Kazakhstan as the main subject for her Ph.D. thesis.

